# Suitability of Alternative Protein Foods for Agroecological Approaches to Address Nutrition in Low- and Middle-Income Countries

**DOI:** 10.1016/j.cdnut.2023.101998

**Published:** 2023-10-30

**Authors:** Molly Gordon, Alexis N. Peña, Ty Beal, Rachel Bezner Kerr

**Affiliations:** 1 Center for Cell Dynamics, Department of Cell Biology, Johns Hopkins School of Medicine, Baltimore, MD, United States; 2 Translational Tissue Engineering Center, Department of Biomedical Engineering, Johns Hopkins School of Medicine, Baltimore, MD, United States; 3 Knowledge Leadership, Global Alliance for Improved Nutrition, Washington, DC, United States; 4 Department of Global Development, Cornell University, Ithaca, NY, United States

**Keywords:** alternative proteins, macroalgae, edible insects, legumes, cultivated meat, agroecology, plant-based meats

## Abstract

Agroecology has been proposed as a holistic approach to transform food systems that meet global food requirements with favorable environmental and social impacts. Agroecology relies on science, practices, and social movements that emphasize ecological principles, local knowledge, culture, and traditions to increase the sustainability and equity of the food system. Agroecological practices have demonstrated positive outcomes on food security and nutrition in low- and middle-income countries (LMICs). Agroecology principles can be applied across the food system and could facilitate the integration of certain alternative protein (AP) foods to address multiple issues. In this perspective, agroecological principles were analyzed to compare the suitability of different AP sources: unprocessed/minimally processed legumes, plant-based meats, edible insects, macroalgae (seaweed), fungal biomass, and cultivated meat. Considerations were identified for the feasibility of AP adoption in LMICs within an agroecological framework to provide nutrient-rich and sustainable diets while addressing other principles such as fairness and economic diversity. From this analysis, legumes, simplified plant-based meat analogs such as texturized plant proteins with minimal additives, edible insects, and macroalgae (location dependent) would make excellent nutritional contributions alongside animal-sourced food within LMICs within an agroecological framework. In contrast, highly processed plant-based meats, fungal biomass, and cultivated meat do not align well with agroecological principles for large-scale human consumption within LMICs. Furthermore, the production facilities to make these foods require robust capital investment and there may be issues related to who owns the intellectual property of these technologies. The NOVA classification system categorizes food based on the degree of processing. Our assessment suggests that foods with lower NOVA classification of unprocessed and minimally processed best fit the agroecological principles related to nutrition, agroecosystem, and societal demands for sustainable food systems.

## Introduction

There is increasing difficulty to meet global sustainable development goals (SDGs), including nutritional requirements linked to SDG2, a trend which will be exacerbated with climate change and as the population climbs to >9 billion people by 2050 [[1], [2], [3]]. Furthermore, the global food system does not provide adequate, safe, and healthy food equitably. Low- and middle-income countries (LMICs) are especially susceptible to climate change impacts on the food system [1] and reportedly have the lowest intake levels of health-promoting foods and the highest levels of malnutrition [3]. Consumption of ultraprocessed foods is rising in LMICs and requires attention to prevent an unhealthy nutrition transition as countries develop.

Animal-sourced foods (ASFs) are rich in nutrients that are commonly lacking globally, including iron, zinc, calcium, vitamins B12 and D, choline, EPA, DHA, and essential amino acids [4]. Imbalanced diets with excess amounts of ultraprocessed foods, processed meat, red meat, and saturated fat can increase risk for noncommunicable diseases [4]. Demand for ultraprocessed foods and ASFs is expected to increase in LMICs (https://foodsystemsdashboard.org). Furthermore, unsustainable industrial livestock production, which supports high levels of meat consumption, leads to widespread biodiversity loss and ecosystem degradation [4]. Although livestock can play an essential role in circular and diverse agroecosystems when produced at appropriate scales in accordance with local ecosystems and sociocultural contexts, alternative protein (AP)-rich foods can also support a transition to sustainable food systems [4]. However, some APs (such as many plant-based meats) use the same unsustainable industrial monoculture systems to produce the raw ingredients, and more sustainable systems are warranted. Full assessment of AP in terms of environmental, social, and nutritional outcomes is thus needed to determine the implications of their use for sustainable food systems.

Agroecology is a holistic approach aiming to optimize ecological processes, environmental and public health, and well-being while minimizing socio-ecological costs from agriculture and food systems [2]. There is increasing evidence and interest in agroecological approaches as a strategy to provide resilience, promote and protect agrobiodiversity, improve soil health, and support communities [5,6]. Thirteen agroecological principles identified by the UN promote shorter food chains, self-sufficiency, sustainability, and healthy and diverse diets, and may play a key role in ensuring adequate nutrition among LMICs [5]. The principles include recycling, input reduction, soil health, animal health, biodiversity, synergy, economic diversification, co-creation of knowledge, social values and diets, fairness, connectivity, land and natural resource governance, and participation [7]. A short description of the 13 principles is provided in Table 1.

**TABLE 1:** Description of agroecological principles (adapted from Wezel et al. [7])

Agroecological principle	Short description
Recycling	Use local renewable materials
Input reduction	Reduce reliance on purchased inputs
Soil health	Support soil health, especially with organic matter and soil biota
Animal health	Improve animal welfare and reduce unnecessary suffering
Biodiversity	Promote diversity of plants and animals at multiple scales
Synergy	Design farming system that supports ecological interaction, complementarity, and synergy between animals, plants, soil, and water and adapts to changing climates
Economic diversification	Promote multiple streams of revenue for small producers
Co-creation of knowledge	Support co-creation and sharing of context-specific knowledge between scientists and local producers
Social value and diets	Support diverse, healthy, seasonally appropriate diets based on local culture and traditions and which are gender equitable
Fairness	Support robust and dignified livelihood for all players, including fair trade, treatment and employment
Connectivity	Build trust and proximity between producers and consumers through short distribution networks
Land and natural resource governance	Ensure responsible governmental processes that allow fair use of natural and genetic resources especially for small-scale producers
Participation	Support social organizations to promote local decision making and management of agricultural and food systems

Agroecological practices embrace animal and plant farming, both of which contribute to a balanced diet. There is, however, a wide range in the frequency of consumption of ASFs within LMICs, from <1 serving/d in South Asia to 5 servings/d in Central Asia [8]. This range, combined with the generally higher economic cost and environmental impact of conventional methods of animal agriculture compared with many plant-sourced foods, suggests that increasing ASF production may not be the ideal path to improved nutrition across LMICs. Several AP sources such as traditional legumes and grains, if produced appropriately, may be key in ensuring adequate nutrition globally because of their potential low cost, availability, and high nutritional value. The food production scale is important to consider as ASF can be produced sustainably though there are limits in scale. For example, natural grassland ecosystems can support integrated crop-livestock systems. Animals are important within the agroecological system and provide nutrients to the soil through manure, eating pests, and plowing fields. Livestock production is also an important source of livelihood, wealth, sociocultural significance, and risk reduction for many small and mid-scale pastoralist and mixed farming systems [6]. Thus, AP must coexist with sustainable livestock production to support livelihoods while mitigating excessive meat consumption, environmental degradation, and high ASF demand.

AP sources can range from traditional foods such as lentils or tofu to novel meat alternatives such as plant-based burgers or cultivated meat. Products can range from unprocessed natural forms to ultraprocessed products such as Impossible™ or Beyond™ burgers. Evaluating the food production chain provides insights into production, processing, and distribution requirements, with agricultural intensification associated with misalignment in agroecological principles. Given the complexity of different AP foods, including their processing levels, the products discussed below were grouped by the NOVA classification. The NOVA system classifies foods based on 4 processing levels with the following descriptive criteria for categories 1–4 [9]: *1*) unprocessed and minimally processed foods, *2*) culinary ingredients, *3*) processed foods, and *4*) ultraprocessed foods. In this perspective, the potential for the current AP landscape to fit within agroecological practices was assessed as a solution to meet livelihood, nutritional needs, and other relevant food system goals across LMICs with minimal environmental impact. We also consider contexts in which APs can serve as viable alternatives to traditional livestock feed, which in turn support culturally appropriate nutritional requirements of the humans who eat them.

## Methodological Approach: Applying Agroecological Principles to the Alternative Protein Landscape

Analysis was conducted to investigate to what extent the following 6 AP sources fit within agroecological principles: unprocessed and minimally processed legumes (beans, peas, and lentils), plant-based meat, edible insects, macroalgae (seaweed), fungal biomass, and cultivated meat (see Supplemental Table 1). Agroecological principles are stratified by 3 ecological scales: agroecosystem, landscape, and the food system. An agroecosystem is composed of both abiotic and biotic components that interact with the physical and chemical environment that are used to produce food for human consumption. The landscape scale refers to the spatial and temporal patterns and zones in a region that can influence the ecosystem. The food system refers to all the inputs and outputs related to food production, processing, and consumption. Food systems consist of complex, heterogeneous, and dynamic food supply chains and food environments.

## Legumes

Legumes and their edible seeds, known as pulses, offer a viable complement or alternative (with careful dietary attention) to ASFs because of their nutritional value. For example, soybeans are one of the only plant-based sources containing all essential amino acids in similar densities and bioavailability as ASF [4]. Complete proteins are foods that contain adequate amounts of all 9 essential amino acids. Most legumes do not satisfy this requirement. To overcome this limitation, legumes are typically complemented with cereal grains to supply all essential amino acids and provide a complete protein meal. Furthermore, nutrition can be enhanced via the sprouting, fermenting, or soaking process, which improves amino acid availability and reduces antinutritional components such as phytic acid. Exogenous phytase can be added to speed up the breakdown of phytic acid. This process relies on the production or import of enzymes and is less aligned with the agroecological principle of input reduction. Legumes are often eaten as whole foods or minimally processed into flours or pastas making them healthy, nutrient-dense crops.

Farming practices for legumes fit well within agroecological principles. Legumes are often grown in mixed-cropping systems with cereals and can be integrated into crop-livestock systems, which contribute to soil health via nitrogen fixation and reduce reliance on the import of chemical fertilizers that may otherwise harm the environment [10]. Crop rotation between legumes and cereals helps deter pests that target specific crops and thus reduces pesticide reliance. Legumes and their coproducts are also nutrient-dense livestock feed [11]. As such, the growth of legumes alongside animals constitutes a synergistic system in which manure from animals can assist soil fertilization for the growth of these crops, which can then go into the animal fodder. Given that ASFs are often a culturally important and nutrient-dense part of local diets, to align with agroecological principles, depending on the context it may be important to find ways to sustainably rear livestock, and locally growing legumes as fodder would be a viable option to reduce input.

Although legumes are enticing alternatives to ASFs, access and control over seed use is of concern. A prominent example is soybean farming, a highly concentrated industry with 80% of the world’s soybeans being produced by just 3 countries [12], the majority of which is grown with proprietary seeds, with only 4 companies controlling 60% of the proprietary seed industry (IPES-Food 2017). Not only does this make soybean production not align with agroecological principles such as input reduction and local governance of genetic resources, but it also leaves LMICs susceptible to supply chain disruptions. Limitations on the use of proprietary soybean seeds because of intellectual property laws make farmers dependent on a highly concentrated industry with a few powerful players (IPES-Food 2017). Most countries have signed agreements that restrict farmers’ rights to reuse seeds based on intellectual property laws [13]. Considering the challenges with the use of soybeans, other legumes can be grown to meet multiple goals. Adaptation of legumes in agroecosystem and sociocultural context would require an agroecological approach for meeting nutrition requirements in LMICs locally, for principles 12 (land and natural resource governance), 5 (biodiversity), and 9 (social values and diets). Pigeon peas, for example, may be suitable depending on climate, as they are a source of fodder, fuelwood, shade, and food; do not have any seed reuse regulation; and their growth promotes soil health via a deep root system that can access otherwise inaccessible nutrients and retain more water, and can be intercropped with groundnuts and maize [14,15]. In other contexts, legumes such as cowpea, common bean, or chickpea might be better suited for the agroecosystem and social context, with heritage foodways also meeting important cultural values. Importantly, farm practices can be shared between farmers, facilitating co-creation and sharing of knowledge [16].

## Plant-based meats

Increased income combined with marketing tactics, a concentrated food industry, and subsidies that reduce the cost of meat production all support shifting dietary preferences that increase demand for ASF [17]. In contrast, growing concerns about environmental and health impacts from meat consumption may influence consumer acceptance toward embracing legumes as major protein sources; but taste and other aspects of desirability are also important [18]. A possible solution is to transform these products into plant-based meat analogs to mimic the texture and taste of ASF, which could improve acceptance in adopting these crops as major protein sources.

The major protein sources in plant-based meats come from plant proteins such as soy, pea, and mung beans. Legume production for large-scale processing into plant-based meats is most often done in intensive monocultures and thus is associated with the detrimental environmental costs of this mode of farming. In some regions (for example, Brazil) soy production can displace tropical forests [19]. In addition, there will be a lower economic diversity for farmers if 1 crop is used as the main protein source.

Unlike the processed texturized plant proteins, ultraprocessed plant-based meats are produced to replace meat in a fast-food context, with several manufacturing processes and added ingredients such as binders, oils, and salt. Research suggests that there are nutritional differences in these products compared with ASFs. For example, key metabolites such as creatinine, hydroxyproline, and anserine were absent from plant-based burgers [20]. A simple approach for promoting plant-based meats that are more aligned with agroecological principles would be to disseminate knowledge of how to produce simplified plant-based meats using local inputs such as protein isolates, heat, and water. Several LMICs have burgeoning industries for the production of protein concentrates and isolates which can be used directly in the generation of texturized meat analogs, for example, in Ethiopia [21]. The resulting meat analogs can be grouped into NOVA classification 3 if the process requires no electricity and simplified tools to generate or classification 4 if industrial machinery is needed. Much of the equipment required for milling and separating proteins from legumes is relatively affordable and accessible and thus can be acquired and used by small businesses or community organizations. Jobs in plant-based meat production facilities could represent an improvement in social value for laborers, because jobs in slaughterhouses are often considered among the most difficult and dangerous of labor roles, including low pay, exploitation, and risk of injury, infection, or psychological distress [22].

## Edible insects

Approximately 2000 insects are part of traditional diets and have varied nutritional content because of abiotic and biotic factors. Broadly, edible insects have a macronutrient profile consisting mostly of proteins and lipids and can be highly micronutrient dense. Edible insect products could be categorized in NOVA group 1 or 2 depending on the final food product as either processed whole or extracted protein, fat, or chitin. Traditional processing methods differ depending on species and may include gut emptying, removal of inedible parts, washing, boiling, or roasting, then subsequent sun drying [23]. Some of these traditional processing methods can improve the taste and texture, and extend the shelf life. Newer processing methods include high-pressure processing and modified atmosphere packaging, and hermetic sealing process can help minimize risk of foodborne pathogen contamination and extend shelf life [24]. Insects are currently farmed largely from natural habitats, and wild harvesting has not been economically scaled. Scaling wild harvesting is undesirable as over-collecting can compromise biodiversity, use of forestry resources, and the insects’ environment while impacting accessibility to people who eat the insects traditionally, which would be contrary to agroecological principles on ensuring biodiversity and resource governance [25]. Insect cultivation is a viable option; it requires limited land and water resources and thus would result in input reduction for high-quality protein sources. Direct emissions of greenhouse gasses in several measured cultivated insects were lower than that of conventional livestock [26]. Insect farming can occur indoors in nonspecialized locations such as in schools for educational purposes, garages, or sheds, which facilitates the spread and co-creation of knowledge for this farming practice [27]. Because of the flexibility of nonspecialized systems, insect cultivation can occur in different climates.

Insect-sourced protein can be cultivated on organic waste side stream feed. Further food safety research to evaluate waste streams to grow insects is needed, because municipal biowaste and manure pose risks for food safety, which is associated with allergenicity [28]. Insects affect soil health and promote synergy because insect feces can be used as organic fertilizer by adding nutrients to the soil and are easily assimilated by plant tissues [29]. In addition, edible agricultural pests that harm crops can be directly harvested from agricultural landscapes and provide economic diversification if sold. Harvesting pests instead of treating them with pesticides reduces purchased inputs while reducing the pesticide residue on food crops themselves and decreasing the amount of hazardous chemical runoff from pesticides [30].

Cultural acceptance of insects varies where insect consumption is not traditional. Continued development of products from insects into familiar products such as flours and powders to promote acceptability and desirability will be critical for adoption. In addition, other approaches such as social marketing and demand generation will be needed to increase consumer interest and demand. The use of edible insects (for example, black soldier fly) as livestock feed could also increase the sustainability of livestock production, particularly for monogastrics and ruminants that compete with human edible food [31].

## Macroalgae (seaweed)

Edible aquatic macroalgae (seaweeds) can be classified as either red, brown, or green seaweed based on photosynthetic pigment composition and are nutrient-dense foods with high dietary fiber and micronutrients and varying protein content by species [32]. Macroalgae fall into NOVA group 1 or 2 depending on the final product with developments in products and ingredients including pasta, soups, snacks, sheets, and condiments. Macroalgae agroecosystems do not require the occupation of arable land and thus leave land available for crops and animals that improve soil quality. Wild harvest requires coastal cities, limiting it to certain regions and may have ecological consequences if harvested in high amounts.

Currently, most macroalgae for food production are cultivated in coastal farming systems. Broad adoption to landlocked countries would require intensive transportation and thus long supply chains. Long supply chains are susceptible to disruptions, limiting resiliency and do not align with agroecological principles related to input reduction, connectivity of producers and consumers, and participation of local communities in food systems. There are examples of aquaculture production and onsite cultivation and use of freshwater macroalgae whereby farm waste nutrients are recycled and used as input sources for the cultivation of phototrophic organisms such as macroalgae [33]. Macroalgae can be recycled and used as a biofertilizer and a conditioning agent.

An important consideration in macroalgae farming is whether to rely on wild harvest of native species or cultivating native and nonnative species. Both farming types can have unintended adverse effects on the ecosystem. For example, wild harvest of native algae species may impact biodiversity by reducing food sources for aquatic creatures. Cultivating algae often relies on intensive monocultures which can negatively affect the nutrient availability of the aquatic ecosystem. Overall, macroalgae as an AP source would be useful in agroecological systems but only for coastal LMICs and upon considering proper harvesting techniques that do not harm the given aquatic ecosystem.

## Fungal biomass

Several species of edible fungi produce large quantities of mycoprotein, which is a complete protein source, and contains high levels of fiber and micronutrients, making it an attractive supplement or ASF replacement [34]. From an agroecosystem standpoint, fungal biomass can be cultivated on low-value waste and byproducts from the food industry and some fungi can promote recycling within a system by using organic waste side streams as a carbon source for growth [35]. Fungal biomass production tends to be ultraprocessed as mycoprotein into products such as burgers, which works against several agroecological principles, such as input reduction and recycling and creates barriers for adoption in LMICs. It not only requires specific infrastructure such as temperature-controlled bioreactors but also requires a second set of machinery for generating the burgers. These capital and energy-intensive steps mean that input use is high, and it reduces the connectivity between producers and consumers. The ultraprocessed foods produced may not be easily adoptable into traditional cuisines, going against the agroecological principle of ensuring foodways support social values in diets. Fungal biomass can also be used as a source of fishmeal, an industry that relies heavily on and disrupts wild fish stock [35]. As such, adopting fungal biomass production within an LMIC agroecological system could include supporting farmers or local cooperatives to install small to mid-size bioreactors to grow fungal biomass that can be processed for local livestock fodder.

## Cultivated meat

Cultivated meat refers to the production of animal tissues outside the animal, which may offer a nutritionally equivalent replacement for meat although more studies are required for validation [36]. Currently, the costs associated with building facilities and supplying cell culture media are prohibitive to most countries for scaling up cultivated meat production. Although some progress has been made to reduce the cost of cell culture media [37], one possible immediate agroecological route for LMICs would be adopting hybrid farming systems in which livestock is still produced for ASF, but also used for genetic and cellular materials as input for cultivated meat processes, through farmer-run cooperatives that ensure fair payment, technical and capital support. This scenario would increase economic diversity, addressing fairness and securing livelihoods for farmers who can contribute to novel technology development without the prohibitive production costs. If costs decline and consumer interest for cultivated meat increases locally, it is also possible that small farms could install their own bioreactors to produce cultivated meat directly alongside their livestock also used for traditional ASFs, which favors animal welfare by reducing total livestock needs [22]. This scenario would require workers with a different skill set to be recruited to these farms to manage the bioreactors, which would ultimately create jobs and foster connectivity, satisfying multiple agroecological principles. Debates remain about cultivated meat from a social value perspective with one roadblock to consumer acceptance of cultivated meat relating to cultural foodways that center around animal hunting, slaughter, and meat butchering such as producing kosher or halal meats [38].

In conclusion, the transformation of the global food system is interdisciplinary, spanning from changes in production practices and technological innovation to dietary shifts. Agroecology is one approach to positively transform the food system [5]. There are examples of successful agroecological systems in LMICs. Studies have found that agroecological practices can have positive outcomes on food security, and there is growing evidence they can have a positive impact on nutrition [2,5]. ASFs are an excellent nutritional source, but global demand for ASF is increasing at a rate that is expected to accelerate environmental destruction and climate change. As such, an agroecological approach that marries AP sources with a sustainable scale of ASF will be the most streamlined path forward to ensure meeting SDG2 with minimal environmental impact.

In this article, 13 agroecological principles were applied to 6 APs to determine the agroecological suitability of each food source in contributing to nutrition goals within LMICs. From this analysis, legumes, simplified plant-based meat analogs such as texturized plant proteins with minimal additives, edible insects, and macroalgae (location dependent) would make excellent nutritional contributions alongside ASF within LMICs within an agroecological framework. To the contrary, highly processed plant-based meats, fungal biomass, and cultivated meat do not align well with agroecological principles for large-scale human consumption within LMICs. Furthermore, the production facilities to make these foods require robust capital investment, and there may be issues related to who owns the intellectual property of these technologies. Our assessment suggests that foods with lower NOVA classification of unprocessed and minimally processed best fit the agroecological principles related to nutrition, agroecosystem, and societal demands for sustainable food systems.

## Author contributions

The authors’ responsibilities were as follows—all authors: contributed to the conceptual design of the article; MG, ANP: wrote the manuscript; TB, RBK: edited drafts; and all authors: read and approved the final manuscript.

## Funding

The Sight and Life Foundation provided funding to support the open access charges for this special supplement.

## Conflict of interest

The authors report no conflicts of interest.

## Appendix A Supplementary data

Multimedia component1 Multimedia component1
