# Supplementary material for: Suitability of Alternative Protein Foods for Agroecological Approaches to Address Nutrition in Low- and Middle-Income Countries
Source: Curr Dev Nutr. 2023 Oct 30;8(Suppl 1):101998. doi: 10.1016/j.cdnut.2023.101998 (PMC10926127; doi:10.1016/j.cdnut.2023.101998)
Supplement: Multimedia component1 [file mmc1.docx]

**Supplemental Table 1.** Summary of suitability of different alternative protein sources and agroecological principles.

|  | **Edible Insects** | **Macroalgae (seaweed)** | **Legumes** | **Cultivated meat and meat analogues** | **Plant-based protein products (pea, wheat, soy)** | **Fungi (biomass)** |
| --- | --- | --- | --- | --- | --- | --- |
| **NOVA Classification*** | Unprocessed (eating live insects) or minimally processed (cricket flour) | Unprocessed (eating dried plants) | Unprocessed (dried beans), minimally processed (lentil flour), processed (lentil pasta) | Ultra-processed (soy-based meat-analogue) | Ultra-processed (cultivated meat cuts) | Ultra-processed (beef patty from fungus) |
| **(1) Recycling** - promoting reuse of materials | Can convert low-value organic by-products into high-value proteins [1].  Requires less water and land and higher energy consumption compared to conventional livestock [2, 3] | Potential to inhibit eutrophication and mitigates ocean acidification and hypoxia. Supports bioremediation through carbon sequestration and terrestrial nutrient runoff absorption. | By-products can be used to make food products for human consumption or used as feedstock for fungal biomass production. | Cell culture products like ammonia can be repurposed to create ammonium sulfate, which can be used as a fertilizer | Mostly leguminous crops used to make protein isolates for plant-based meats | Some fungal species can efficiently grow on organic-rich waste streams such as vinasse or potato protein liquor [4] |
| **(2) Input reduction -** less reliance on imports | In mixed crop-livestock systems, use of insects as livestock feed or insect waste as a biofertilizer reduces reliance other inputs such as synthetic inputs [5] | Freshwater macroalgae can be cultivated using nutrient wastewater from freshwater fish farming or farm waste nutrients which can aid in bioremediation and biomass production [6]  Macroalgae can be used as livestock foodstuffs [7, 8] | Pulse crop residue can be used as nutritious fodder for cattle, reducing the need to import more feed [9] | May increase external inputs due to the number of food-grade components needed for the cell culture media | Similar to legumes section | Because fungal biomass can be processed as animal feed, this would reduce the need to import food for current livestock. |
| **(3) Soil health -** improving soil fertility | Insect feces can be used as an organic fertilizer to promote soil health and plant growth [10] | Brown algal extracts are used to increase the productivity of a variety of agricultural plants [11] Macroalgal forests stabilize coastal sediments and erosion control [12] | Growth of various legumes enhance soil quality through nitrogen fixation in mixed crop systems. | Impact on soil health would be an indirect effect of having less livestock farms | Similar to legumes with the exception that if soy is used as the major protein-source this is not typically grown in mixed crop systems but rather as intensive monocultures. | Impact on soil health would be an indirect effect of having less livestock farms |
| **(4) Animal health -** improve animal welfare and reduce unnecessary suffering | Insects can be an alternative to fish feed used in some livestock systems thereby potentially mitigating harmful fishing practices. | Algae can improve aquatic animal health such as protecting mollusks under ocean acidification scenarios [13] | Pulse crop residue can be used as nutritious fodder for cattle | Reduces reliance on traditional animal agriculture by providing slaughter-free meat alternatives. | Reduces reliance on traditional animal agriculture by providing slaughter-free meat alternatives. | Reduces reliance on traditional animal agriculture by providing protein-rich alternatives |
| **(5) Biodiversity -** promote a healthy mixture of plants and animals | Insects are mainly harvested from natural habitats, which must be properly regulated to prevent loss of biodiversity. | Harvesting algae directly from kelp forests can risk disturbing biodiversity [14].  Considerations for fish spawning grounds and nurseries are needed. | Densely flowering legumes support rich pollinator assemblages [15]. | Reduces land use by 63%, 72%, and 81-95% compared to conventional chicken, pork, and beef production, respectively. Land can be rewilded to preserve biodiversity and sequester carbon [16]. | If monocrops like soybeans are used, this poses a risk to biodiversity as the soils may not be easily used by other plants and animals. | As with cultivated meat and plant-based meat, fungal biomass meat analogues could divert need for farmland that can be restored to promote biodiversity. |
| **(6) Synergy** -design of a diversified farming system that uses local flora and fauna and adapts to changing climates | Commercial farming has challenges like potential spread of pathogens. | Co-culture of marine algae improves health of some aquatic species such as promoting survival of shrimp infected with certain viruses [17]. | Can improve crop production and diseases through intercropping [18]. | Less infectious disease including zoonotic infectious disease due to reduced meat products in markets | Certain crops used for plant-based meats such as peas are cover crops, which can improve soil in between growing seasons for cash crops by protecting the soil from erosion and nutrient loss. | Can be used as an alternate source of fishmeal, which is currently strongly dependent on the capture and over-exploitation of wild fish stock. |
| **(7) Economic diversification** - promote multiple streams of revenue for small producers | Edible insects that are considered pests to a local agricultural system can be harvested for food [19]. | Algae used in bioremediation of fish wastewater are often commercially valuable for example as agar-agar or food sources for high value fish such as abalone. | Side streams from pulse protein processing can be used to generate alternative revenue sources such as aquafaba and pulse fiber [20]. | Possibility for cattle farmers to earn additional income by providing sources of genetic material for cell cultured meat. | If farmers to reduce cattle on their land, one potential revenue stream for landowners could be payments (e.g., from governments) for ecosystem services such as carbon sequestration or biodiversity conservation generated by habitat restoration. | Possibility to install bioreactors on farms for farmers to produce fungal biomass for fishmeal to supplement income. |
| **(8) Co-creation of knowledge** - context-specific knowledge being spread between local producers | Small-scale farming in a school setting can supplement protein sources, provide educational opportunities, and stimulate market demand [21]. | Seaweed cultivation in Zanzibar is labor intensive and done mostly by women. Traditional medicine preparations from macroalgae are considered professional secrets and has known uses as organic fertilizer. Fishermen have more knowledge than female seaweed farmers [22]. | Heterogeneity of crop management and soil fertility require participatory co-design process to develop relevant farming systems with options and out-scaling [23]. | Agri-tourism could develop around farm bioreactors with opportunities to learn about the cultured meat production process [24]. | Generation of simplified textured plant proteins can be easily passed between people. | Alternative uses for fungal biomass is an area of active research for example, fungal-induced flocculation of algae. This technique has implications in wastewater treatment and biofuel production [25]. |
| **(9) Social value and diets** - emphasis on diversity, equity, and inclusion while improving health and livelihood of local communities | ~2000 edible insects are established parts of traditional diets globally. | Traditionally eaten in some Asian countries and can provide recreation, historical, aesthetic, spiritual, and religious values [22]. | Central part of the diet for many communities around the world [26].  Anti-nutrients such as phytic acid may hinder nutrient absorption. Cannot assume levels reported on dietary labels provide sufficient nutrients for level of absorption. | Must consider cultural practices involving the hunting, processing, and eating of the animal.  There is a lack of studies comparing traditional meat to cultivated meat with respect to nutrition [27]. | This sector lags behind in the production of true cuts of meat and mostly involves ground meat substitutes. This limits the applications with respect to certain cultural cuisines.  Varies substantially with respect to nutrition compared to animal meat. | Usually heavily processes the product into nuggets or patties, limiting the use in traditional cuisine.  Mycoprotein is high in protein and fiber and low in energy and saturated fat and contains no trans-fat or cholesterol. |
| **(10) Fairness** - support robust and dignified livelihood for all players | Can improve livelihood through diversified income, strengthening human and social capital [28]. | Unknown implication for increased use of wild stocks, there is a need to develop large-scale and cost-effective algae cultivation methods to meet growing demand [8, 29]. | Lower profitability than other crops like cereals [26]. | Less fair because technology is inaccessible to the average person. | Required equipment is relatively affordable and can be installed at farms to facilitate production from the source. | Highly specialized skills and facilities limits participation in this technology. |
| **(11) Connectivity** - closing the gap from producer to consumer | Current insect farming is primarily done at small-scale. | Important for local rural coastal communities [22, 30]. | Some legumes, like pigeon pea is a smallholder crop that is consumed locally | If farmers transition to produce cultivated meat, consumer trust may be increased as food is coming from the same trusted source | Some texturized plant-proteins can be made with small equipment installed in farms. |  |
| **(12) Land and natural resource governance** - responsible governmental orders to allow fair use of natural resources | There are community monitoring programs to conserve species and wild habitats [31]. Established food safety legislation on insect rearing practices is needed. | Require proper coastal water regulation. There are many national and international conventions and policies to protect coastal macroalgae habitats. | Most soy is from genetically modified seed that is controlled by a few select companies within a highly distorted industry. | Only land required is for production facilities. | Similar concerns to legumes, for example governance over soybean use. | Only land required is for production facilities. |
| **(13) Participation** - support social organization to promote local decision-making in food production | Small insect farms can be employed in a variety of spaces enabling a democratized protein source. Rural farmers can employ additional workers to harvest edible pest insects. | Support of many marine sector actors or employ multi-trophic systems to ensure economic feasibility (fish, shellfish, and seaweed) [32]. | Farming practices and techniques can be shared between farmers. | Highly specialized skills and facilities limits participation in this technology. | Generation of simplified textured plant proteins can be easily passed between people. | Highly specialized skills and facilities limits participation in this technology. |
| *NOVA is a food classification system that categorizes foods according to the extent and purpose of food processing rather than in terms of nutrients. There are four groupings according to the nature, extent, and purpose of industrial food processing used in their production. Group 1 designates unprocessed or minimally processed foods, group 2 includes processed culinary ingredients, group 3 is processed foods, and group 4 includes ultra-processed food and drink products. | | | | | | |

References for Supplemental Table:

1. Manurung, R., Supriatna, A., Esyanthi, R.R. and Putra, R.E, *Bioconversion of rice straw waste by black soldier fly larvae (Hermetia illucens L.): optimal feed rate for biomass production.* J Entomol Zool Stud, 2016. **4**: p. 1036-1041.

2. Oonincx, D.G. and I.J. de Boer, *Environmental impact of the production of mealworms as a protein source for humans - a life cycle assessment.* PLoS One, 2012. **7**(12): p. e51145.

3. Smetana, S., et al., *Sustainability of insect use for feed and food: Life Cycle Assessment perspective.* Journal of Cleaner Production, 2016. **137**: p. 741-751.

4. Karimi, S., et al., *Use of Organic Wastes and Industrial By-Products to Produce Filamentous Fungi with Potential as Aqua-Feed Ingredients.* Sustainability, 2018. **10**(9).

5. Mier y Terán Giménez Cacho, M., et al., *Bringing agroecology to scale: key drivers and emblematic cases.* Agroecology and Sustainable Food Systems, 2018. **42**(6): p. 637-665.

6. Cole, A.J., R. de Nys, and N.A. Paul, *Removing constraints on the biomass production of freshwater macroalgae by manipulating water exchange to manage nutrient flux.* PLoS One, 2014. **9**(7): p. e101284.

7. Overland, M., L.T. Mydland, and A. Skrede, *Marine macroalgae as sources of protein and bioactive compounds in feed for monogastric animals.* J Sci Food Agric, 2019. **99**(1): p. 13-24.

8. Costa, M., et al., *Current knowledge and future perspectives of the use of seaweeds for livestock production and meat quality: a systematic review.* J Anim Physiol Anim Nutr (Berl), 2021. **105**(6): p. 1075-1102.

9. Sherasia, P.L., Manget Ram Garg, and B. M. Bhanderi., *Pulses and their by-products as animal feed.* 2018.

10. Poveda, J., *Insect frass in the development of sustainable agriculture. A review.* Agronomy for Sustainable Development, 2021. **41**(1).

11. Hamed, S.M., et al., *Role of marine macroalgae in plant protection & improvement for sustainable agriculture technology.* Beni-Suef University Journal of Basic and Applied Sciences, 2018. **7**(1): p. 104-110.

12. Bos, A.R., et al., *Ecosystem engineering by annual intertidal seagrass beds: Sediment accretion and modification.* Estuarine, Coastal and Shelf Science, 2007. **74**(1-2): p. 344-348.

13. Fernández, P.A., P.P. Leal, and L.A. Henríquez, *Co-culture in marine farms: macroalgae can act as chemical refuge for shell-forming molluscs under an ocean acidification scenario.* Phycologia, 2019. **58**(5): p. 542-551.

14. Campbell, I., et al., *The Environmental Risks Associated With the Development of Seaweed Farming in Europe - Prioritizing Key Knowledge Gaps.* Frontiers in Marine Science, 2019. **6**.

15. Cole, L.J., et al., *Supporting wild pollinators in agricultural landscapes through targeted legume mixtures.* Agric Ecosyst Environ, 2022. **323**: p. 107648.

16. Swartz, E., *Anticipatory Life Cycle Assessment and Techno‐Economic Assessment of Commercial Cultivated Meat Production: A Summary of Recommended Stakeholder Actions.* The Good Food Institute, 2021.

17. Anaya-Rosas, R.E., et al., *Effects of a co-culture of marine algae and shrimp (Litopenaeus vannamei) on the growth, survival and immune response of shrimp infected with Vibrio parahaemolyticus and white spot virus (WSSV).* Fish Shellfish Immunol, 2019. **87**: p. 136-143.

18. Semba, R.D., Ramsing, R., Rahman, N., Kraemer, K. and Bloem, M.W., , *Legumes as a sustainable source of protein in human diets.* Global Food Security, 2021. **28**: p. 100520.

19. Cerritos, R.a.K., M., *Pre-Hispanic agricultural practices: Using pest insects as an alternative source of protein.* Animal Frontiers, 2015. **5**: p. 31-36.

20. Ratnayake, W.S. and S. Naguleswaran, *Utilizing side streams of pulse protein processing: A review.* Legume Science, 2021. **4**(1).

21. Hanboonsong, Y., Tasanee Jamjanya, and Patrick B. Durst, *Six-legged livestock: edible insect farming, collection and marketing in Thailand.* RAP publication 3, 2013: p. 8-21.

22. de la Torre-Castro, M. and P. Rönnbäck, *Links between humans and seagrasses—an example from tropical East Africa.* Ocean & Coastal Management, 2004. **47**(7-8): p. 361-387.

23. Ronner, E., et al., *Co-design of improved climbing bean production practices for smallholder farmers in the highlands of Uganda.* Agric Syst, 2019. **175**: p. 1-12.

24. Newton, P. and D. Blaustein-Rejto, *Social and Economic Opportunities and Challenges of Plant-Based and Cultured Meat for Rural Producers in the US.* Frontiers in Sustainable Food Systems, 2021. **5**.

25. Muradov, N., et al., *Fungal-assisted algal flocculation: application in wastewater treatment and biofuel production.* Biotechnol Biofuels, 2015. **8**: p. 24.

26. Alvarez, M.M.H., *Health benefits of pulses.* 2019.

27. Fraeye, I., et al., *Sensorial and Nutritional Aspects of Cultured Meat in Comparison to Traditional Meat: Much to Be Inferred.* Front Nutr, 2020. **7**: p. 35.

28. Halloran, A., et al., *Insects in the human food chain: global status and opportunities.* Food Chain, 2014. **4**(2): p. 103-118.

29. van Oirschot, R., et al., *Explorative environmental life cycle assessment for system design of seaweed cultivation and drying.* Algal Research, 2017. **27**: p. 43-54.

30. Moberg, F., and Carl Folke, *Ecological goods and services of coral reef ecosystems.* Ecological economics, 1999. **29**: p. 215-233.

31. Musundire, R., et al., *Stewardship of Wild and Farmed Edible Insects as Food and Feed in Sub-Saharan Africa: A Perspective.* Front Vet Sci, 2021. **8**: p. 601386.

32. Cerca, M., A. Sosa, and F. Murphy, *Responsible supply systems for macroalgae: Upscaling seaweed cultivation in Ireland.* Aquaculture, 2023. **563**.
